# Management of Severe Respiratory Failure after Acute Empyema Surgery in a Patient with Down Syndrome: A Case Report

**DOI:** 10.70352/scrj.cr.25-0667

**Published:** 2026-04-01

**Authors:** Chisaki Ichinohe, Kengo Tani, Daisuke Kimura, Tsubasa Matsuo, Takahiro Sasaki, Shuta Kimura, Masahito Minakawa

**Affiliations:** 1 Department of Thoracic and Cardiovascular Surgery, Hirosaki University Graduate School of Medicine, Hirosaki, Aomori, Japan; 2 Department of Thoracic Surgery, Akita University Graduate School of Medicine, Akita, Akita, Japan

**Keywords:** acute empyema, Down syndrome, respiratory failure, atelectasis, inhaled nitric oxide, intrapulmonary percussive ventilation, prone positioning therapy

## Abstract

**INTRODUCTION:**

Empyema associated with parapneumonic pleural effusion can cause respiratory failure requiring surgical intervention. However, some cases remain difficult to manage even after surgery. Patients with Down syndrome are particularly susceptible to respiratory infections and tend to develop more severe symptoms. Few studies have addressed the management of postoperative respiratory failure in acute empyema.

**CASE PRESENTATION:**

A 12-year-old girl with Down syndrome developed acute empyema following right-sided parapneumonic pleural effusion and underwent thoracoscopic empyema cavity debridement. Surgical intervention alone was not sufficient to resolve postoperative respiratory failure. She was managed with adjunctive therapies including inhaled nitric oxide (iNO), intrapulmonary percussive ventilation (IPV), and prone positioning therapy. These therapies resulted in marked improvements in postoperative atelectasis and respiratory failure, enabling successful weaning from mechanical ventilation.

**CONCLUSIONS:**

The combined use of iNO, IPV, and prone positioning therapy may be an effective management strategy for severe respiratory failure after acute empyema surgery in patients with Down syndrome. These adjunctive therapies could contribute to recovery of respiratory function in patients with postoperative atelectasis.

## Abbreviations

ARDS
: acute respiratory distress syndrome
CRP
: C-reactive protein
FIO_2_
: fraction of inspiratory oxygen
iNO
: inhaled nitric oxide
IPV
: intrapulmonary percussive ventilation
PaO_2_
: partial pressure of oxygen
PCV
: pressure control ventilation
WBC
: white blood cell

## INTRODUCTION

Pleural effusion is observed in approximately 20%–40% of pneumonia cases, and empyema develops in approximately 5%–10% of patients.^1–3)^ Chest tube drainage, antibiotics, and fibrinolytic agents are recommended in cases of acute empyema. However, if these treatments fail and pleural collection persists, surgical intervention is usually required.^4)^ Although surgical intervention reduces the rates of complications and mortality compared with chest tube drainage alone,^5,6)^ a considerable number of cases remain challenging to manage.

Patients with severe physical and intellectual disabilities, such as those with cerebral palsy or with Down syndrome, are more susceptible to respiratory infections and more likely to develop severe symptoms compared with healthy individuals. In particular, patients with Down syndrome have an increased incidence of respiratory tract infections, which may be associated with congenital heart disease, abnormal airway anatomy and physiology, hypotonia, and aspiration.^7)^

We encountered a patient with Down syndrome who developed acute empyema following parapneumonic pleural effusion. As surgical treatment alone was insufficient, the patient was successfully managed with adjunctive therapies including iNO, IPV, and prone positioning therapy.

## CASE PRESENTATION

**Fig. 1:**
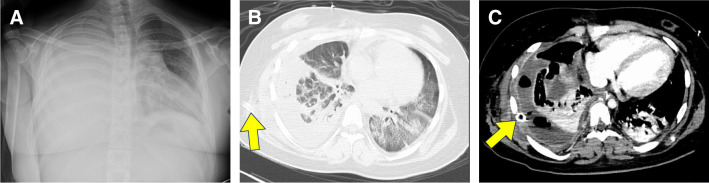
Preoperative findings. The drainage tube is shown as a yellow arrow. (**A**) Chest radiograph at the referring hospital shows pleural effusion occupying the entire right lung with deviation of the trachea and mediastinum. (**B**) CT after right chest drainage reveals infiltrative shadows in both lung fields, leading to a diagnosis of parapneumonic pleural effusion. (**C**) CT on the eighth day of hospitalization reveals thickening of the visceral pleura and multiloculated abscess cavities, leading to the diagnosis of acute empyema.

A 12-year-old girl with Down syndrome presented with fever of 38°C and cough. She had a history of ventricular septal defect and patent ductus arteriosus repair at 5 months of age, as well as hypothyroidism and hyperuricemia. Oral medication was initially prescribed by her primary care doctor, and later she visited a pediatrician at her referring hospital because of worsening respiratory distress. Chest radiography revealed a large right-sided pleural effusion, which led to hospital admission (**Fig. 1A**). Despite chest drainage, only 300 mL of fluid was removed. Her respiratory condition deteriorated; intubation and mechanical ventilation were required. Due to severe respiratory failure, a PaO_2_ of 51.7 mmHg was observed in an FIO_2_ of 100%. The patient was transferred to the pediatric department of Hirosaki University Hospital. Chest CT revealed infiltrative shadows in both lung fields (**Fig. 1B**). She was diagnosed with ARDS secondary to severe pneumonia and admitted to the ICU. The laboratory findings on admission were: WBC 21650/μL, hemoglobin 9.0 g/dL, creatinine 1.68 mg/dL, and CRP 45.6 mg/dL. Sputum cultures and blood cultures were negative.

The ventilator was set to PCV, with an inspiratory pressure of 18 cmH_2_O and a positive end-expiratory pressure of 15 cmH_2_O. The pediatrician initiated antibiotics, iNO, and continuous hemodiafiltration. Although her general condition temporarily improved (PaO_2_ 97.1 mmHg, FiO_2_ 40%, WBC 11030/μL, CRP 2.5 mg/dL), she developed a fever of 38.6°C and had an elevated inflammatory response (PaO_2_ 73.3 mmHg, FiO_2_ 40%, WBC 15,670/μL, CRP 28.5 mg/dL) 1 week after admission. A follow-up chest CT was performed, and acute empyema was suspected (**Fig. 1C**). Consequently, the patient was referred to our department where emergency thoracoscopic-assisted right empyema cavity debridement was performed.

**Fig. 2:**
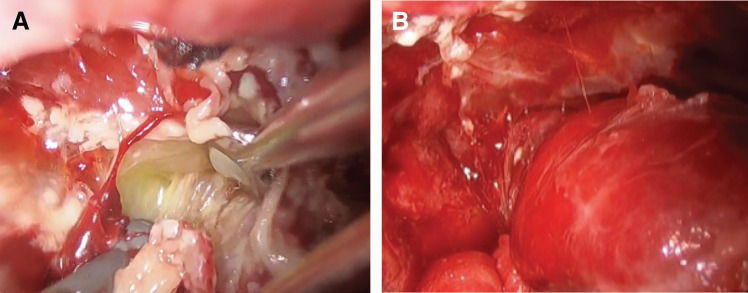
Intraoperative findings. (**A**) Before debridement. The abscess cavity is multiloculated and fibrinopurulent. Cultures tested negative for pathogens. (**B**) After debridement. The abscess cavity is converted into a single chamber. Due to insufficient oxygenation, one-lung ventilation could not be performed adequately.

A bronchial blocker was inserted and the patient was placed in the left lateral position. Due to poor oxygenation, surgery was performed with intermittent bilateral lung ventilation. The chest drain inserted through the sixth intercostal space was removed and the wound was enlarged to 5 cm. The abscess cavity had become multichambered and was in the fibrinopurulent phase (**Fig. 2**). An additional port was created in the eighth intercostal space. The septum was scraped to unify the abscess cavity. The pleural space was irrigated with 10000 mL of saline and the surgery was completed with 2 drains in place. Both pleural plaque and fluid cultures tested negative for pathogens.

**Fig. 3:**
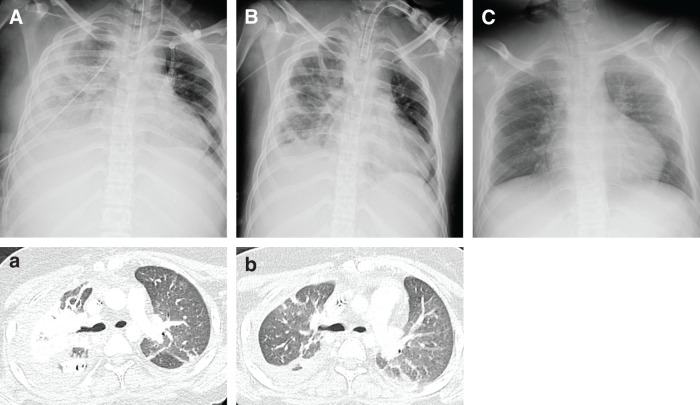
Time-course changes in chest imaging. (**A**, **a**) Chest radiograph (**A**) and CT image (**a**) obtained on POD 6 reveal right-sided atelectasis. (**B**, **b**) Chest radiograph (**B**) and CT image (**b**) obtained after IPV and prone positioning therapy demonstrate improvement of right-sided atelectasis. Tracheostomy was performed because of prolonged mechanical ventilation. (**C**) Chest radiograph obtained on POD 41 shows further improvement after successful weaning from mechanical ventilation. IPV, intrapulmonary percussive ventilation

**Fig. 4:**
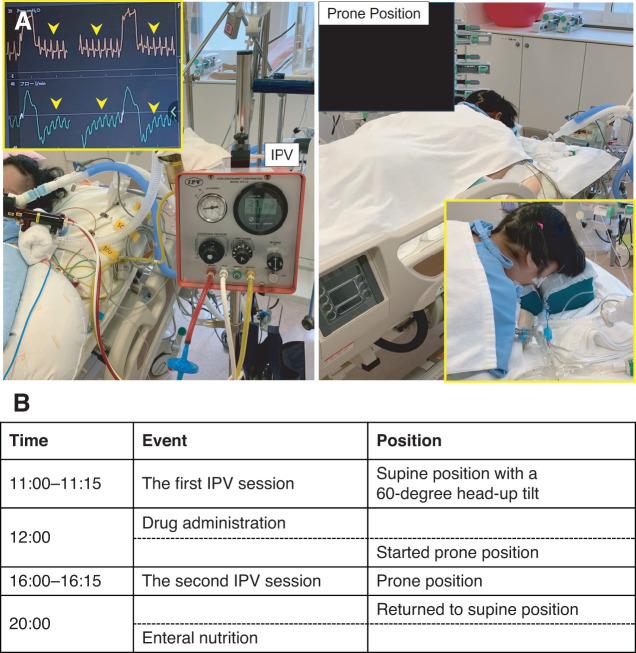
IPV and prone positioning therapy. (**A**) IPV was performed for 15 minutes per session, with a maximum airway pressure of 20 cmH_2_O and a percussion frequency of 240 cycles per minute. The yellow arrowheads indicate the percussion waveforms. (**B**) The daily schedule of IPV and prone positioning. IPV, intrapulmonary percussive ventilation

**Fig. 5:**
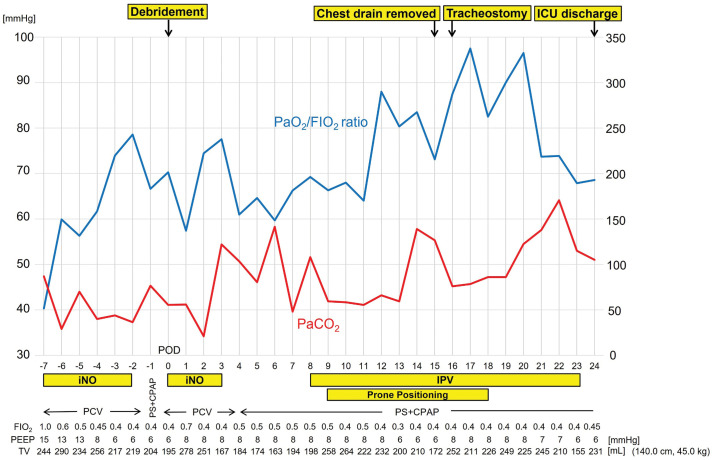
Clinical course from admission to ICU discharge. FIO_2_, fraction of inspiratory oxygen; iNO, inhaled nitric oxide; PaCO_2_, partial pressure of carbon dioxide; PaO_2_, partial pressure of oxygen; PCV, pressure control ventilation; PEEP, positive end-expiratory pressure; PS + CPAP, pressure support with continuous positive airway pressure; TV, tidal volume

iNO therapy was resumed and continued until POD 3. On POD 4, the ventilator mode was changed from PCV to pressure support with continuous positive airway pressure; however, right-sided atelectasis became apparent, resulting in worsening oxygenation (**Fig. 3A**). Bronchoscopy revealed a moderate amount of viscous sputum; however, suctioning failed to improve oxygenation (PaO_2_ 74.2 mmHg, FIO_2_ 50%) or resolve the atelectasis. Therefore, IPV (IPV-1C; Percussionaire Japan, Tokyo, Japan) and prone positioning therapy were initiated (**Fig. 4**). IPV was started on POD 8 and continued for 16 days, with a maximum airway pressure of 20 cmH_2_O, a percussion frequency of 240 cycles/minute, for 15 minutes per session, twice daily. Prone positioning therapy was initiated on POD 9. The chest drain was removed on POD 15, and a tracheostomy was performed on POD 16. Following the initiation of IPV and prone positioning therapy, enhanced sputum clearance led to resolution of the obstructive atelectasis (**Fig. 3B**). Sputum cultures and blood cultures were repeated periodically, and all results remained negative. Considering the planned transfer to a general ward, the development of mandibular epidermal exfoliation and pressure ulcers, and the improvement of atelectasis, prone positioning was discontinued on POD 18. She was discharged from the ICU on POD 24. The clinical course from admission to ICU discharge is shown in **Fig. 5**. The antibiotic therapy continued until POD 46. The patient was weaned from mechanical ventilation on POD 41 (**Fig. 3C**), and the tracheostomy cannula was removed on POD 119.

## DISCUSSION

For patients with acute empyema that does not improve with drainage alone, early surgical intervention is recommended.^4–6)^ However, some cases remain difficult to manage, even after surgery. In our patient, surgery successfully controlled the empyema infection; although, postoperative atelectasis developed, and surgery alone was insufficient to improve her respiratory status. The patient was saved with the combined use of iNO, IPV, and prone positioning therapy.

NO is a smooth muscle relaxant produced by vascular endothelial cells that can selectively dilate pulmonary blood vessels, which has traditionally been used in the management of neonatal hypoxic respiratory failure and persistent pulmonary hypertension.^8)^ Owing to its ability to reduce pulmonary hypertension and improve oxygenation, it is sometimes administered as an adjunctive life-saving measure or to enhance oxygenation in patients with ARDS.^8,9)^ In recent years, iNO has been utilized in the treatment of acute bronchiolitis and as a rescue therapy for severe pneumonia caused by coronavirus disease 2019.^9,10)^ However, improvements in oxygenation are typically transient and few studies have demonstrated that iNO improves the overall prognosis of ARDS.^11)^ Therefore, iNO should be regarded as an adjunctive therapy for refractory hypoxemia rather than as a definitive therapy for respiratory failure. In Japan, iNO is approved and reimbursed only for persistent pulmonary hypertension of the newborn and for use during the perioperative period of cardiovascular surgery. Nevertheless, owing to its ability to reduce pulmonary hypertension and improve oxygenation, iNO is sometimes used off-label as an adjunctive life-saving measure in patients with ARDS.^12)^ In the present case, iNO was administered off-label to achieve a temporary improvement in oxygenation.

In this patient, IPV and prone positioning therapy were effective in managing the atelectasis and respiratory failure caused by postoperative sputum retention. IPV is a modification of intermittent positive pressure ventilation that delivers percussive bursts of gas into the airways at a frequency of 60–600 breaths per minute, synchronized with the patient respiratory cycle.^13)^ By inducing internal oscillations in the lungs, IPV promotes fluidization and the subsequent ejection of airway secretions. As demonstrated in this case, IPV was particularly effective for obstructive atelectasis caused by sputum accumulation. Compared with conventional respiratory physiotherapy, it offers more rapid improvement in atelectasis cases.^14)^ IPV can be utilized not only in endotracheally intubated patients but also through various interfaces such as mouthpieces, facemasks, and tracheostomy tubes.^15,16)^

IPV has been proven effective in the treatment of a wide range of diseases, ranging from acute to chronic conditions. It is applicable to patients of all ages, from children to the older adults, including those who have difficulty clearing sputum independently.^15,17)^ Recent studies have reported the effectiveness of IPV in managing respiratory failure in patients with severe physical and intellectual disabilities, demonstrating its potential as a valuable therapeutic option for this vulnerable population.^18)^ In individuals with Down syndrome, anatomical abnormalities, immunodeficiency, and related complications increase susceptibility to respiratory infections and contribute to their severity.^7,19)^ In addition, hypotonia and reduced muscle strength in patients with Down syndrome impair effective coughing and hinder airway clearance, potentially leading to sputum retention. We believe that IPV is an effective treatment for severe atelectasis and respiratory failure in patients with Down syndrome, as demonstrated in the present case.

Prone positioning therapy is recommended as a lung-protective ventilation strategy for respiratory failure associated with ARDS.^20–22)^ Alveolar units are more numerous in the dorsal lung than in the ventral lung. In the supine position, the dorsal lung is compressed by gravity, making the ventral alveoli more easily expandable. Consequently, alveolar collapse occurs more readily in the dorsal region, leading to atelectasis. Pulmonary blood flow is greater in the dorsal lung due to gravitational effects. This results in a greater degree of ventilation-perfusion mismatch, leading to poor oxygenation. In the prone position, gravitational effects on lung compression are altered, reducing ventral lung overinflation and promoting more uniform ventilation throughout the lung. Importantly, pulmonary blood flow distribution does not change substantially with positional changes; in both the supine and prone positions, perfusion consistently predominates in the dorsal regions. Thus, in the prone position, ventilation of the dorsal lung improves while perfusion is preserved, resulting in improved gas exchange.^20)^ The prone position promotes ventilation in the dorsal lung regions, increases the number of alveoli participating in gas exchange, and reduces intrapulmonary shunting. During mechanical ventilation, increasing positive end-expiratory pressure in the supine position may cause overdistension in less-injured lung regions; by contrast, prone positioning promotes more uniform ventilation throughout the lungs and improves lung recruitment.^21)^

During therapy, ensuring sufficient staffing for safe repositioning is essential, especially in patients with impaired head control or those under sedation, while carefully monitoring for potential disconnection or displacement of the vascular lines and endotracheal tube.^21)^ Given the planned transfer to a general ward, where staffing levels are lower than in the ICU, and the improvement in atelectasis, prone positioning was discontinued on POD 18. Thereafter, regular positional changes were actively encouraged.

In this case, postoperative atelectasis was improved by combining prone positioning therapy and IPV. Prone positioning enhanced ventilation and alveolar recruitment in the dorsal lung regions, while IPV facilitated sputum clearance, resulting in synergistic improvement in oxygenation and resolution of obstructive atelectasis. To our knowledge, there have been no previous reports on the combined use of prone positioning therapy and IPV. Although this is a single case, our findings suggest that prone positioning therapy may augment the effectiveness of IPV, and that other therapeutic modalities could potentially be more effective when used in combination with prone positioning.

## CONCLUSIONS

A patient with acute empyema and severe respiratory failure who was unresponsive to chest drainage alone underwent thoracoscopic-assisted debridement of the empyema cavity. However, surgical intervention alone was insufficient to achieve adequate respiratory management. iNO was administered to temporarily improve oxygenation. Postoperative respiratory failure due to extensive atelectasis was successfully managed using prone positioning therapy and IPV.
